# Fli1-haploinsufficient dermal fibroblasts promote skin-localized transdifferentiation of Th2-like regulatory T cells

**DOI:** 10.1186/s13075-018-1521-3

**Published:** 2018-02-07

**Authors:** Ryosuke Saigusa, Yoshihide Asano, Takashi Taniguchi, Megumi Hirabayashi, Kouki Nakamura, Shunsuke Miura, Takashi Yamashita, Takehiro Takahashi, Yohei Ichimura, Tetsuo Toyama, Ayumi Yoshizaki, Maria Trojanowska, Shinichi Sato

**Affiliations:** 1 Department of Dermatology, University of Tokyo Graduate School of Medicine, 7-3-1 Hongo, Bunkyo-ku, Tokyo, 113-8655 Japan; 2 Arthritis Center, Boston University School of Medicine, Boston, MA USA

**Keywords:** Fli1, Fibroblasts, Regulatory T cells, IL-33, Systemic sclerosis

## Abstract

**Background:**

Friend leukemia virus integration 1 (Fli1) deficiency, a predisposing factor of systemic sclerosis (SSc), induces SSc-like phenotypes in various cell types. A recent study demonstrated the transdifferentiation of T helper type 2 cell (Th2)-like regulatory T cells (Tregs) in SSc lesional skin through interleukin (IL)-33 produced by fibroblasts. Therefore, we investigated the role of Fli1 deficiency in dermal fibroblast-mediated transdifferentiation of Tregs.

**Methods:**

Cytokine expression was assessed in Tregs by flow cytometry and in skin samples and cultivated cells by immunostaining, immunoblotting, and/or qRT-PCR. Fli1 binding to the target gene promoters was examined by chromatin immunoprecipitation. Murine dermal fibroblasts and Tregs were cocultured with or without blocking antibodies against target cytokines.

**Results:**

Th2- and Th17-like cell proportions in skin-homing Tregs were increased in bleomycin-treated *Fli1*^*+/−*^ mice compared with bleomycin-treated wild-type mice, whereas Th1-, Th2-, and Th17-like cell proportions in splenic Tregs were comparable. *Fli1*^+/−^ fibroblasts overproduced IL-33 and IL-6, in particular IL-33, and Fli1 occupied the *IL33* and *IL6* promoters in dermal fibroblasts. Importantly, the IL-4-producing cell proportion was significantly higher in wild-type Tregs cocultured with *Fli1*^+/−^ fibroblasts than in those cocultured with wild-type fibroblasts, which were canceled by neutralizing anti-IL-33 antibody. Under the same coculture condition, an increased tendency of IL-17A-producing cell proportion, which was possibly mediated by IL-6, was evident.

**Conclusions:**

Fli1 haploinsufficiency increases the proportions of Th2- and Th17-like Tregs in bleomycin-induced profibrotic skin conditions, in which IL-33-producing dermal fibroblasts contribute to Th2-like Treg transdifferentiation, suggesting a critical role of Fli1 deficiency in the interaction of dermal fibroblasts with immune cells in pathological skin fibrosis.

**Electronic supplementary material:**

The online version of this article (10.1186/s13075-018-1521-3) contains supplementary material, which is available to authorized users.

## Background

Systemic sclerosis (SSc) is a multisystem autoimmune disease characterized by vasculopathy and fibrosis of the skin and certain internal organs. Although SSc pathogenesis has yet to be fully disclosed, the canonical wisdom is that dermal fibroblast activation is the final consequence of its sequential pathological processes, such as initial vascular injury due to autoimmune attack and subsequent chronic inflammation [1]. However, a recent study has demonstrated that activated fibroblasts regulate tissue-localized transdifferentiation of regulatory T cells (Tregs) into T helper type 2 cell (Th2)-like cells through IL-33 in SSc lesional skin [2], suggesting that activated dermal fibroblasts amplify an aberrant immune response characteristic of SSc. This notion provides new insight into the continuing discussion of the controversy of Tregs in SSc, with reports of decreased, increased, or equal proportions of Tregs in the blood of patients compared with healthy control subjects [3–6]. Although Treg transdifferentiation into Th1- and/or Th17-like cells has been reported in type 1 diabetes [7], multiple sclerosis [8], and juvenile idiopathic arthritis [9], Treg transdifferentiation into Th2-like cells is documented exclusively in SSc so far, indicating that this pathological process is unique in this disease.

Friend leukemia virus integration 1 (Fli1) is a member of the Ets family of transcription factors, the deficiency of which is a potential predisposing factor of SSc [10]. Fli1 expression is suppressed in dermal fibroblasts, endothelial cells, and perivascular inflammatory cells in involved and noninvolved skin of patients with SSc [11]. Although the detailed mechanism explaining Fli1 downregulation in SSc is still unknown, an epigenetic mechanism is reported at least in dermal fibroblasts [12]. According to a series of studies on *Fli1*-mutated mice and *FLI1* small interfering RNA-treated cultured cells, Fli1 deficiency promotes the induction of SSc-like phenotypes in dermal fibroblasts, dermal microvascular endothelial cells, and macrophages [13–16]. Most importantly, mice with simultaneous haploinsufficiency of the *Fli1* and *Klf5* genes, both of which are epigenetically suppressed in SSc dermal fibroblasts, spontaneously develop the three cardinal features of SSc, including immune abnormalities, vasculopathy, and tissue fibrosis [17]. These animal models are useful for obtaining a clue to understanding the role of certain cells and to elucidating the mechanisms of disease-modifying drugs in SSc [18, 19].

On the basis of this background, we investigated the role of Fli1 deficiency in fibroblast-mediated transdifferentiation of Tregs by using bleomycin (BLM)-treated *Fli1*^+/−^ mice. The detailed molecular mechanism was further investigated by coculture experiments of Tregs with dermal fibroblasts.

## Methods

### A BLM-induced murine SSc model

BLM (200 μg, 02907278; Nippon Kayaku, Tokyo, Japan) dissolved in PBS or control PBS was injected subcutaneously into a single location on the back of 8-week-old female wild-type (WT) mice (C57BL/6) and *Fli1*^+/−^ mice daily.

### Flow cytometry

Mice were treated with BLM for 1 week. On the day after the final injection, lymphocytes from the spleen and the dermis of the lower back skin were obtained. Skin samples were incubated in 2 mg/ml dispase (383-02281; Wako Pure Chemical Industries, Osaka, Japan) and were separated into epidermis and dermis. Dermis was minced and then incubated with 2 mg/ml collagenase type 2 (CLS-2; Worthington Biochemical, Lakewood, NJ, USA) in Tyrode’s solution for 60–90 minutes. The digested tissues were centrifuged, resuspended in PBS, and filtered through a 70-μm mesh. Single-cell suspensions were stained on ice with labeled monoclonal antibody. In the surface staining experiments, cells were stained with antibodies against CD4 (100526; BioLegend, San Diego, CA, USA). In intracellular cytokine staining, cells were stimulated with 10 ng/ml phorbol myristate acetate and 1 μg/ml ionomycin (10634; Sigma-Aldrich, St. Louis, MO, USA) in the presence of 1 mg/ml brefeldin A (420601; BioLegend) for 4 h. Cells were washed; stained for CD4; treated with fixative/permeabilization buffer (71-5775-40; eBioscience, San Diego, CA, USA); and then stained with anti-FoxP3 (71-5775-40; eBioscience), anti-IL-4 (554436; BD Biosciences, San Jose, CA, USA), anti-IL-13 (12-7133-41; eBioscience), anti-IL-17A (506919; BioLegend), and anti-interferon (IFN)-γ (505825; BioLegend) antibodies. Tregs were defined by CD4^+^FoxP3^+^ cells. Cells were analyzed on a FACSVerse flow cytometer (BD Biosciences).

### Immunohistochemistry

Immunohistochemistry with the Mouse on Mouse (M.O.M^TM^) Elite Peroxidase Detection Kit (PK-2200; Vector Laboratories, Burlingame, CA, USA) was performed on formalin-fixed, paraffin-embedded skin sections using anti-IL-33 antibody (ab54384; Abcam, Cambridge, UK). To quantify the signal intensity of IL-33, color images were converted to grayscale, and then the brightness was measured in five different randomly selected fibroblasts and epidermal areas per specimen. These processes were analyzed using ImageJ software (National Institutes of Health, Bethesda, MD, USA), and the average values were compared.

### Murine dermal fibroblast isolation

For isolation of murine fibroblasts, referring to a past report [20], we took the ears from mice and placed them in cell culture media. Then, collagenase (17100017; Thermo Fisher Scientific, Waltham, MA, USA) was added, and the ears were minced as much as possible with sterile scissors. After overnight incubation at 37 °C, the ears were further dissociated by vigorous pipetting. Cells were passed through a cell strainer to achieve a single-cell suspension and remove the remaining debris. Finally, the cells were pelleted by centrifugation, resuspended, and cultured until proliferated.

### qRT-PCR

RNA isolation from cultivated cells and skin tissue, as well as qRT-PCR, was performed as described previously [21–23]. The sequences of primers were as follows: murine *Il33*-forward 5′-CAATCAGGCGACGGTGTGGATGG-3′, murine *Il33*-reverse 5′-TCCGGAGGCGAGACGTCACC-3′; murine *Il6*-forward 5′-GATGGATGCTACCAAACTGGAT-3′, murine *Il6*-reverse 5′-CCAGGTAGCTATGGTACTCCAGA-3′; murine *Gapdh*-forward 5′-CGTGTTCCTACCCCCAATGT-3′, murine *Gapdh*-reverse 5′-TGTCATCATACTTGGCAGGTTTCT-3′.

### Immunoblotting

Confluent quiescent fibroblasts were serum-starved for 24 h. Whole-cell lysates were prepared. Samples were subjected to sodium dodecyl sulfate-PAGE (NP0321; Life Technologies/Thermo Fisher Scientific, Carlsbad, CA, USA) and immunoblotting with anti-IL-33 antibody (ab54384; Abcam) and anti-β-actin antibody (sc-47778; Santa Cruz Biotechnology, Dallas, TX, USA). Bands were detected using enhanced chemiluminescence techniques (34080; Thermo Fisher Scientific, Rockford, IL, USA).

### Chromatin immunoprecipitation assay

Human normal dermal fibroblasts were purchased from the American Type Culture Collection (PCS-201-010; Manassas, VA, USA) and used. The chromatin immunoprecipitation (ChIP) assay was conducted using the EpiQuik ChIP kit (P-2002-3; EpiGentek, Farmingdale, NY, USA) [17]. Putative Fli1 binding sites in the *IL33* and *IL6* promoters were predicted using Tfsitescan. The primers that amplify fragments of the *IL33* promoter (−1332 bp to approximately −1183 bp) and the *IL6* promoter (−1109 bp to approximately −944 bp) were as follows: *IL33* ChIP-forward 5′-TCAGCTGGGAGATGGGTAAG-3′; *IL33* ChIP-reverse 5′-ATAATCTATTCTCTCTGAAGCCTACAA-3′, *IL6* ChIP-forward 5′-GACACCATCCTGAGGGAAGA-3′; *IL6* ChIP-reverse 5′-TATCGCTCCCTCTCCCTGTA-3′. In some experiments, fibroblasts were treated with IL-1β (201-LB-005; R&D Systems, Minneapolis, MN, USA).

### Cocultures

Murine dermal fibroblasts were prepared from WT and *Fli1*^+/−^ mice and maintained as described previously [13]. Splenic Tregs were isolated from WT mice with a CD4^+^CD25^+^ Treg cell isolation kit (130-091-041; Miltenyi Biotec, Bergisch Gladbach, Germany) and cultured in RPMI 1640 medium supplemented with FCS. Murine dermal fibroblasts (1 × 10^5^ cells) and CD4^+^CD25^+^ T cells (3 × 10^5^ cells) were cocultured in 24-well plates for 2 days. Then, cells were analyzed on a FACSVerse flow cytometer. In some experiments, cocultured cells were treated with anti-mouse IL-33 antibody (M187-3; MBL, Nagoya, Japan) or antimouse IL-6 antibody (MAB406-SP; R&D Systems).

### Statistical analysis

Statistical analysis was done with the Mann-Whitney *U* test to compare the distributions of two unmatched groups. Statistical significance was defined as a *P* value < 0.05.

## Results

### Th2- and Th17-like Tregs are increased in the skin of BLM-treated *Fli1*^*+/−*^ mice

**Fig. 1:**
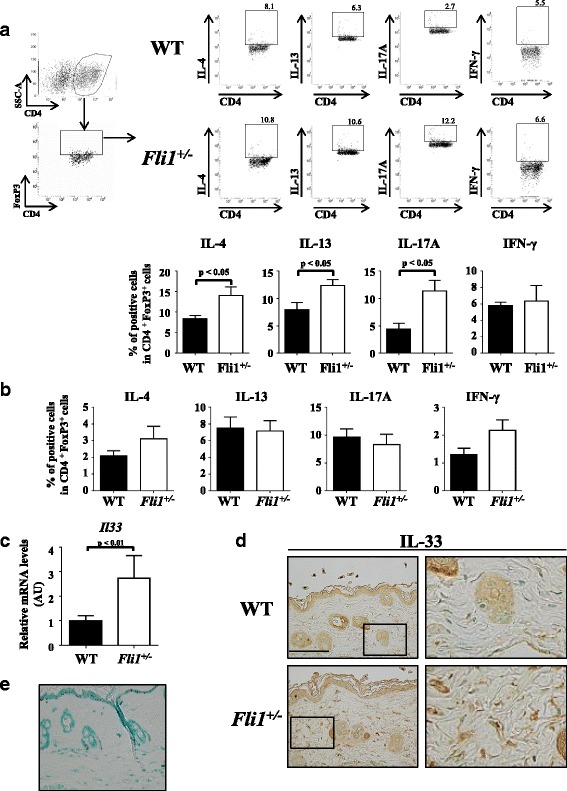
Cytokine expression profiles of skin-homing and splenic regulatory T cells (Tregs) in bleomycin (BLM)-treated mice and interleukin (IL)-33 expression in the lesional skin of BLM-treated mice. **a** Evaluation of skin-homing Tregs from BLM-treated wild-type (WT) and *Fli1*^*+/−*^ mice on IL-4, IL-13, IL17A, and IFN-γ expression by flow cytometry (*n* = 6). Gating strategy for identification of CD4^+^FoxP3^+^ Tregs is shown in the *leftmost panel*. **b** Evaluation of splenic Tregs from BLM-treated WT and *Fli1*^*+/−*^ mice on IL-4, IL-17A, and IFN-γ expression by flow cytometry (*n* = 6). **c** qRT-PCR evaluation of *Il33* messenger RNA (mRNA) expression in the lesional skin of WT and *Fli1*^*+/−*^ mice treated with BLM for 1 week (*n* = 10). **d** Representative images of staining for IL-33 in skin samples from WT and *Fli1*^*+/−*^ mice treated with BLM for 1 week (original magnification ×400, scale bar = 50 μm). **e** The result of immunostaining without anti-IL-33 antibody in the skin of BLM-treated WT mice. Images of high-power field spotlighting dermal fibroblasts are in the *rightmost column*. Representative plots of one of five to eight individual animals from at least two separate experiments are shown in (**a**). In each graph, the relative value compared with the control group is expressed as mean ± SEM. *AU* Arbitrary units, *Fli1* Friend leukemia virus integration 1, *SSC-A* Side scatter area

As an initial experiment, we employed BLM-treated mice because this murine model recapitulates inflammatory and fibrotic aspects of SSc [24]. In this model, inflammation and dermal fibrosis reach their peak at days 7 and 28 after BLM injection, respectively [25–28]. After confirming that BLM induces greater dermal fibrosis in *Fli1*^*+/−*^ mice than in WT mice as previously reported [13], we collected skin samples and splenocytes at day 7 and evaluated the phenotypes of skin-homing and splenic Tregs by comparing the proportions of IFN-γ-, IL-4-, IL-13-, and IL-17A-producing cells among CD4^+^FoxP3^+^ cells. As shown in Fig. 1a, on one hand, BLM injection increased the proportions of IL-4-, IL-13-, and IL-17A-producing Tregs—namely, Th2- and Th17-like Tregs—in the skin of *Fli1*^*+/−*^ mice compared with WT mice. On the other hand, the proportions of Th1-, Th2-, and Th17-like cells in splenic Tregs were comparable between BLM-treated *Fli1*^*+/−*^ mice and BLM-treated WT mice (Fig. 1b; scatterplots also shown in Additional file 1: Figure S1). These results suggest that Fli1 haploinsufficiency promotes skin-localized transdifferentiation and/or skin infiltration of Th2- and Th17-like Tregs in BLM-treated mice.

### IL-33 expression is higher in skin of BLM-treated *Fli1*^*+/−*^ mice

Because IL-33 produced by dermal fibroblasts contributes to Th2-like Treg transdifferentiation in the lesional skin of patients with SSc [2], we next evaluated IL-33 expression in the skin of BLM-treated *Fli1*^*+/−*^ mice. Of note, *Il33* messenger RNA (mRNA) expression was higher in the skin of BLM-treated *Fli1*^*+/−*^ mice than in BLM-treated WT mice (Fig. 1c). To confirm the origin of BLM-induced IL-33 expression, we also carried out immunostaining with anti-IL-33 antibody. As shown in Fig. 1d and Additional file 2: Figure S2, the signal intensity of IL-33 in dermal fibroblasts was remarkably increased in BLM-treated *Fli1*^+/−^ mice as compared with BLM-treated WT mice, whereas that in epidermal keratinocytes was comparable. When the staining was performed without anti-IL-33 antibody, no signals were detectable in the same skin sample from BLM-treated WT mice (Fig. 1e). Taken together, these results indicate that IL-33 produced by dermal fibroblasts may contribute to Th2-like Treg transdifferentiation in the skin of BLM-treated *Fli1*^+/−^ mice.

### Fli1 haploinsufficiency results in overexpression of IL-33 in dermal fibroblasts

**Fig. 2:**
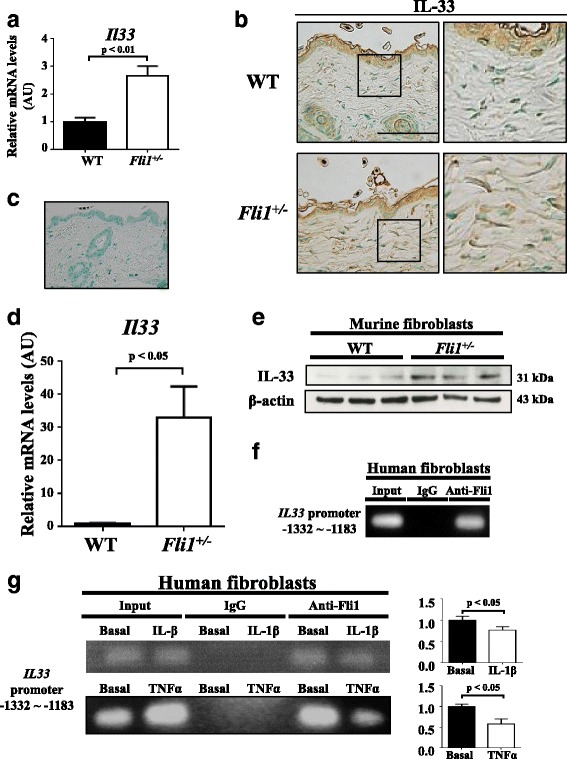
Friend leukemia virus integration 1 (Fli1) regulates the expression of interleukin (IL)-33 in dermal fibroblasts. **a**, **b** Evaluation of IL-33 expression in skin from wild-type (WT) and *Fli1*^*+/−*^ mice without any treatment by qRT-PCR (**a**, *n* = 10) and immunohistochemistry (**b**, *n* = 5; original magnification ×400, scale bar = 50 μm). High-power field images spotlighting dermal fibroblasts are in the *rightmost column*. **c** The result of immunostaining without anti-IL-33 antibody in the skin of WT mice under a physiological condition. **d**, **e** Evaluation of IL-33 expression in dermal fibroblasts isolated from WT and *Fli1*^*+/−*^ mice by qRT-PCR (**d**, *n* = 10) and immunoblotting (**e**, *n* = 4). **f**, **g** Results of the chromatin immunoprecipitation assay regarding Fli1 occupation on the *IL33* promoter in human dermal fibroblasts without any treatment (**f**, *n* = 4) and those cells treated with IL-1β or tumor necrosis factor (TNF)-α for 24 h (**g**, *n* = 4). For **a** and **c**, messenger RNA (mRNA) levels of the *Il33* gene were normalized to those of the *Gapdh* gene. For **f**, the occupancy of the *Il33* promoter by Fli1 was quantified with qRT-PCR. For **b**, **d**, and **e**, representative results are shown. In each graph, the relative value compared with the control group is expressed as mean ± SEM. *AU* Arbitrary units, *IgG* Immunoglobulin G, *TNF-α* Tumor necrosis factor-α

To investigate the molecular mechanism underlying IL-33 overproduction in dermal fibroblasts of BLM-treated *Fli1*^+/−^ mice, we first evaluated IL-33 expression levels in the skin of *Fli1*^+/−^ and WT mice under a physiological condition. Of note, *Il33* mRNA levels were significantly elevated in the skin of *Fli1*^+/−^ mice compared with WT mice (Fig. 2a). Furthermore, IL-33 expression was clearly elevated in dermal fibroblasts of *Fli1*^+/−^ mice relative to those cells of WT mice while being comparable in epidermal keratinocytes (Fig. 2b and Additional file 2: Figure S2). Also, no nonspecific signals were confirmed by conducting staining without anti-IL-33 antibody (Fig. 2c). Importantly, the signal intensity of IL-33 in dermal fibroblasts was modestly but significantly elevated in BLM-treated *Fli1*^+/−^ mice compared with untreated *Fli1*^+/−^ mice (Additional file 2: Figure S2). Supporting these in vivo findings, IL-33 expression was increased in *Fli1*^*+/−*^ dermal fibroblasts at mRNA and protein levels compared with WT dermal fibroblasts in vitro (Fig. 2d and e; *see* Additional file 3: Figure S3 for an original file). These results indicate that *Fli1*^*+/−*^ dermal fibroblasts produce excessive amounts of IL-33 even under a quiescent condition.

To further examine if Fli1 directly regulates the expression of IL-33, we conducted in vitro experiments with human dermal fibroblasts. The ChIP assay exhibited the binding of Fli1 to the *IL33* promoter (Fig. 2f), suggesting the direct regulation of IL-33 by Fli1. Given that IL-1β and tumor necrosis factor (TNF)-α induce the expression of IL-33 in dermal fibroblasts [29], we investigated whether these molecules affect the occupancy of Fli1 on the *IL33* promoter by performing ChIP analysis, showing that both of IL-1β and TNF-α induced the dissociation of Fli1 from the promoter (Fig. 2g). In aggregate, these results indicate that Fli1 directly regulates IL-33 expression as a potent repressor and that Fli1 deficiency possibly contributes to IL-33 production in SSc dermal fibroblasts.

### Transdifferentiation of Tregs into Th2-like cells is promoted by *Fli1*^*+/−*^ dermal fibroblasts through IL-33 in vitro

**Fig. 3:**
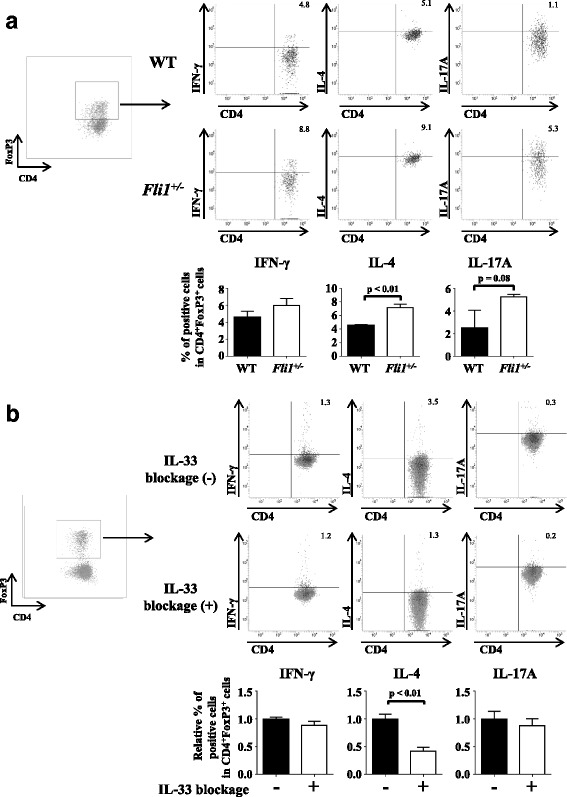
T helper type 2 cell (Th2)-like regulatory T cells (Tregs) are induced by coculture with *Fli1*^*+/−*^ dermal fibroblasts through interleukin (IL)-33. **a** Evaluation by flow cytometry of Th1-, Th2-, and Th17-like Treg induction by coculture with wild-type (WT) and *Fli1*^*+/−*^ dermal fibroblasts (*n* = 6). **b** Evaluation by flow cytometry of the effect of IL-33-neutralizing antibody on Tregs cocultured with *Fli1*^*+/−*^ dermal fibroblasts (*n* = 6). Representative plots of interferon (IFN)-γ-, IL-4-, and IL-17A-positive Tregs are shown in *right upper panels* of (**a**) and (**b**). Gating strategy for identification of CD4^+^FoxP3^+^ Tregs is shown in the *leftmost panels* of (**a**) and (**b**). In each graph, the relative value compared with the control group is expressed as mean ± SEM. *AU* Arbitrary units, *Fli1* Friend leukemia virus integration 1

Because fibroblast-mediated transdifferentiation of Th2-like Tregs depends on IL-33 in SSc, we next assessed if the same scenario is applicable to Fli1 haploinsufficiency-dependent transdifferentiation of Th2-like Tregs in BLM-treated mice. To this end, we cocultured WT Tregs with dermal fibroblasts isolated from WT mice or *Fli1*^+/−^ mice. The proportion of IL-4-producing cells was significantly higher and the proportion of IL-17A-producing cells tended to be higher in CD4^+^FoxP3^+^ cells cocultured with *Fli1*^*+/−*^ dermal fibroblasts than in those cells cocultured with WT fibroblasts, whereas no effect was seen on the proportion of IFN-γ-producing cells (Fig. 3a). On the basis of the data up to this point, Fli1 deficiency in dermal fibroblasts may induce IL-4-expressing Tregs, possibly through the key molecule IL-33. To address this issue, we cocultured these cells in the presence of neutralizing antibody for IL-33. As expected, this neutralizing antibody significantly attenuated the proportion of IL-4-producing cells, whereas it did not affect the proportions of IL-17A- and IFN-γ-producing cells in CD4^+^FoxP3^+^ cells cocultured with *Fli1*^*+/−*^ dermal fibroblasts (Fig. 3b). These results indicate that *Fli1*^*+/−*^ dermal fibroblasts regulate the transdifferentiation of Th2-like Tregs at least partially through IL-33.

**Fig. 4:**
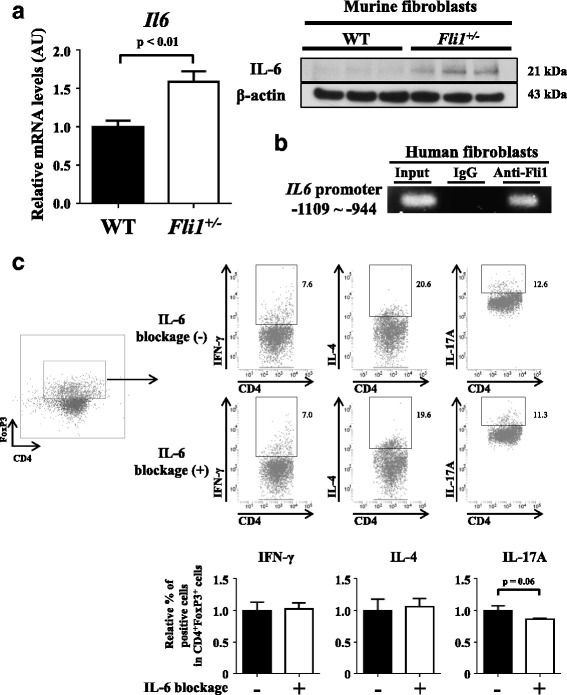
*Fli1*^+/−^ dermal fibroblasts may modestly and partially contribute to the induction of T helper type 17 cell (Th17)-like regulatory T cells (Tregs) by interleukin (IL)-6 production. **a** Evaluation of IL-6 expression in dermal fibroblasts isolated from wild-type (WT) and *Fli1*^*+/−*^ mice (*n* = 10 for each group) by qRT-PCR (*left panel*) and in dermal fibroblasts isolated from bleomycin-treated WT and *Fli1*^*+/−*^ mice (*n* = 3 for each group) by immunoblotting (*right panel*). **b** Results of the chromatin immunoprecipitation assay regarding Fli1 occupation on the *IL6* promoter with human dermal fibroblasts (*n* = 4). **c** Evaluation by flow cytometry of the effect of IL-6 neutralizing antibody on Tregs cocultured with *Fli1*^*+/−*^ dermal fibroblasts (*n* = 6). Representative results of immunoblotting, agarose gel electrophoresis, and flow cytometry are shown for the *right panels* of **a** and **b** and the *right upper panel* of **c**, respectively. Gating strategy for identification of CD4^+^FoxP3^+^ Tregs is shown in the *leftmost panel* of **c**. In each graph, the relative value compared with the control group is expressed as mean ± SEM. *AU* Arbitrary units, *Fli1* Friend leukemia virus integration 1, *IgG* Immunoglobulin G, *mRNA* Messenger RNA

We also assessed whether *Fli1*^+/−^ dermal fibroblasts promote the transdifferentiation of Th17-like Tregs, because a significant increase in the proportion of IL-17A-producing Tregs in the skin of BLM-treated *Fli1*^+/−^ mice (Fig. 1a) and an increased tendency of IL-17A-producing cells in WT Tregs cocultured with *Fli1*^+/−^ dermal fibroblasts (Fig. 3a) were evident. Because IL-6 is a key cytokine in the conversion of induced Tregs into Th17 cells [30, 31] and its mRNA expression is increased in the lesional skin of BLM-treated *Fli1*^+/−^ mice compared with BLM-treated WT mice [13], we speculated that IL-6 produced by *Fli1*^+/−^ dermal fibroblasts promotes the transdifferentiation of Th17-like Tregs. Consistent with this hypothesis, *Il6* mRNA expression was significantly higher in *Fli1*^+/−^ dermal fibroblasts than in WT dermal fibroblasts (Fig. 4a, *left panel*). Also, IL-6 protein expression was elevated in cultured dermal fibroblasts from BLM-treated *Fli1*^*+/−*^ mice compared with those from BLM-treated WT mice (Fig. 4a, *right panel*; *see* Additional file 3: Figure S3 for an original file). The ChIP analysis revealed the binding of Fli1 to the *IL6* promoter in human normal dermal fibroblasts, suggesting the direct regulation of IL-6 expression by Fli1 (Fig. 4b). More importantly, anti-IL-6 antibody tended to reduce the proportion of Th17-like cells in WT Tregs cocultured with *Fli1*^+/−^ dermal fibroblasts (Fig. 4c). Taken together, these results indicate that Fli1 haploinsufficiency increases the proportion of Th17-like cells in skin-homing Tregs, possibly and partially through IL-6 produced by dermal fibroblasts.

## Discussion

BLM-treated mice have been widely used as a useful model of SSc [32, 33]. On one hand, the benefit of this model is the highly reproducible induction of inflammation and tissue fibrosis similar to those of SSc. In addition, it can be used in various genetically manipulated mice to explore the relationship of targeted molecules with inflammatory and fibrotic pathways related to SSc development. On the other hand, the weak point of this model is the lack of persistent tissue fibrosis like that in SSc; namely, tissue fibrosis spontaneously improves within 3 months after ceasing BLM injection. We currently focused on the contribution of Fli1 haploinsufficiency to dermal fibroblast-dependent transdifferentiation of Tregs during the inflammatory phase of an SSc-like condition; therefore, we can say that BLM-treated mice would be suitable for addressing this issue. In our initial experiments, the proportions of Th2- and Th17-like cells in skin-homing Tregs were significantly increased in BLM-treated *Fli1*^+/−^ mice compared with BLM-treated WT mice, which is consistent with our previous report demonstrating the increased expression of Th2 and Th17 cytokines in the lesional skin of BLM-treated mice [13]. Importantly, Th2-like Treg transdifferentiation was regulated at least partially by IL-33 overproduced by *Fli1*^+/−^ dermal fibroblasts. In addition, IL-6 produced by *Fli1*^+/−^ dermal fibroblasts seemed to be modestly involved in Th17-like Treg transdifferentiation. Because Fli1 occupied the *IL33* and *IL6* promoters, Fli1 haploinsufficiency is likely to directly enhance the production of IL-33 and IL-6 in dermal fibroblasts. These results indicate that Fli1 haploinsufficiency augments BLM-induced skin fibrosis by increasing the production of profibrotic Th2 cytokines and the induction of Th17 cytokine-mediated inflammation partially through the interaction of dermal fibroblasts and Tregs.

A critical contribution of Fli1 deficiency to the activation of dermal fibroblasts was initially proved on the basis of its impact on type I collagen expression. Fli1 binds to the *COL1A1* and *COL1A2* promoters [21, 34] and serves as a potent repressor of these genes in dermal fibroblasts [35]. The inverse correlation between Fli1 and type I collagen expression is clearly shown in *Fli1*^+/+^, *Fli1*^+/−^, and *Fli1*^−/−^ murine embryonic fibroblasts [11]. In addition to type I collagen, Fli1 deficiency increases integrin α_v_β_3_ and α_v_β_5_ expression, establishing autocrine transforming growth factor (TGF)-β signaling in dermal fibroblasts [13]. Furthermore, Fli1 deficiency enhances progranulin production, which renders dermal fibroblasts resistant to the antifibrotic effect of TNF-α [36]. In the present study, we proposed a novel notion that Fli1 deficiency regulates skin-localized transdifferentiation of Th2-like Tregs through IL-33 and possibly that of Th17-like Tregs through IL-6, both of which are produced by dermal fibroblasts. Considering that Fli1 deficiency promotes endothelial-to-mesenchymal transition and M2 macrophage differentiation [13], Fli1 deficiency integrates fibrosis-related gene programs toward the profibrotic condition in various types of cells.

Th2-skewed immune polarization has been implicated in the development of SSc-associated tissue fibrosis on the basis of clinical data. In the early stage of diffuse cutaneous systemic sclerosis (dcSSc), serum IL-6 and IL-10 levels are significantly elevated, whereas they are decreased to normal levels in the late stage of dcSSc characterized by the improvement of skin sclerosis [37]. IL-4 remains at normal levels in the early stage of dcSSc, but it is decreased along with the resolution of skin sclerosis. In contrast, serum IL-12 levels are decreased in the early stage of dcSSc, then they gradually increase in parallel with disease duration and finally reach significantly higher than levels normal controls in the late stage of dcSSc [38]. Thus, Th2-skewed immune polarization is quite evident in the early inflammatory phase of dcSSc. Because IL-4 and IL-13 have a profibrotic effect on dermal fibroblasts, such as the induction of type I collagen expression [39], the predominance of Th2 cytokines seems to be involved in the development of tissue fibrosis in SSc. Therefore, Th2-like Tregs may augment BLM-induced dermal fibrosis in *Fli1*^+/−^ mice. In addition, Th2-like Tregs have the potential to induce SSc-like features in other types of cells under a Fli1-deficient condition. For instance, *Fli1*^+/−^ macrophages preferentially differentiate into an M2 phenotype in response to IL-4 stimulation [13]. In endothelial cells, IL-4 suppresses VE-cadherin expression and reduces vascular integrity [40], which is a characteristic feature of SSc vasculopathy. Because Fli1 deficiency downregulates VE-cadherin expression [14], Th2-like Tregs further promote the induction of SSc-like vascular features in BLM-treated *Fli1*^+/−^ mice. Also, *Fli1*^+/−^ dermal fibroblasts overproduce progranulin, which suppresses the antifibrotic effect of TNF-α [36]. Therefore, dermal fibroblasts preferentially respond to the profibrotic stimuli in BLM-treated *Fli1*^+/−^ mice. Thus, Th2-like Tregs possibly induced by IL-33-producing dermal fibroblasts coordinately amplify tissue fibrosis through the phenotypical alteration of various kinds of cells in BLM-treated *Fli1*^+/−^ mice.

In contrast to a significant contribution of IL-33 to *Fli1*^+/−^ dermal fibroblast-dependent Th2-like Treg transdifferentiation, there was just a trend toward the induction of Th17-like Tregs by coculture with *Fli1*^+/−^ dermal fibroblasts and the suppression of *Fli1*^+/−^ dermal fibroblast-dependent Th17-like Treg transdifferentiation by anti-IL-6 antibody. These results suggest that IL-6 produced by dermal fibroblasts modestly and partially contributes to Th17-like Treg transdifferentiation. Indeed, the fold induction of Fli1 haploinsufficiency-dependent expression was much smaller in IL-6 (~ 1.6 times) than in IL-33 (~ 33 times) in dermal fibroblasts. Taken together with the evidence that IL-6 is overproduced by a variety of cells under inflammatory conditions, including endothelial cells, keratinocytes, and inflammatory cells as well as dermal fibroblasts [41, 42], the contribution of dermal fibroblasts to the total amount of IL-6 expression seems to be relatively small in the lesional skin of BLM-treated *Fli1*^+/−^ mice, suggesting a weak contribution of dermal fibroblasts to Th17-like Treg differentiation. However, we need to be aware that IL-6-dependent redifferentiation of Tregs into Th17 cells is dependent on IL-1 and TGF-β [43]. Given that IL-1β is upregulated in the lesional skin of BLM-treated *Fli1*^+/−^ mice [13] and that integrin α_v_β_3_ and α_v_β_5_, which promote the release of active TGF-β from its latent form [44, 45], are upregulated in dermal fibroblasts of BLM-treated *Fli1*^+/−^ mice [13], IL-6 seems to coordinately promote the differentiation of Th17-like Tregs together with IL-1β and TGF-β in the lesional skin of these mice. Therefore, the contribution of fibroblast-derived IL-6 to the development of Th17-like Tregs was likely underestimated in the coculture experiment. Another important point is that, in addition to skin-localized transdifferentiation of Th17-like Tregs, the increased infiltration of circulating Th17-like Tregs into the lesional skin may occur in BLM-treated *Fli1*^+/−^ mice. Relevant to this hypothesis is our previous demonstration that Fli1 deficiency induces the expression of intercellular adhesion molecule-1 and glycosylation-dependent cell adhesion molecule-1, positive regulators for Th2 and Th17 cell infiltration, while suppressing the expression of E-selectin and P-selectin, positive regulators of Th1 cell infiltration [13, 46]. These previous data propose the possibility that the infiltration of circulating Th17-like Tregs into the lesional skin may be facilitated by altered expression of cell adhesion molecules in BLM-treated *Fli1*^+/−^ mice. Further studies are required to clarify these points.

The contribution of IL-17A to the development of skin fibrosis has been well studied in BLM-treated mice. For instance, Th17 cell infiltration and IL-17A expression in the skin are increased and serum IL-17A levels correlate with the severity of skin fibrosis in this murine model [47]. Furthermore, loss of *Il17a*, but not *Ifnγ* and *Il4*, results in the reduction of BLM-induced skin fibrosis and IL-17A stimulation induces the expression of TGF-β and connective tissue growth factor in NIH3T6 fibroblasts [24]. Therefore, IL-17A serves as a potent profibrotic cytokine in skin fibrosis of BLM-treated mice. According to the present data, we speculate that Fli1 haploinsufficiency enhances Th17-like cell proportions in skin-homing Tregs, subsequently amplifying tissue fibrosis through the profibrotic effect of IL-17A on dermal fibroblasts. Given that *Fli1*^+/−^ dermal fibroblasts produce excessive amounts of type I collagen under a physiological condition [11], Fli1 haploinsufficiency likely contributes to a feedforward relationship between dermal fibroblasts and Th17-like Tregs, which underlies extensive skin fibrosis.

In a recent study, MacDonald et al. [2] reported that the proportions of conventional Th17 cells in CD4^+^FoxP3^−^ T cells and Th17-like Tregs in CD4^+^FoxP3^+^ T cells are comparable in the skin of 36 patients with SSc (average disease duration 5.7 ± 1.1 years for dcSSc and 5.7 ± 1.8 years for limited cutaneous systemic sclerosis) and 20 healthy control subjects. In contrast, a significantly increased proportion of Th17-like Tregs in the skin of BLM-treated *Fli1*^+/−^ mice was evident in the present study. This discrepancy seems to be plausible, considering that the activity and severity of profibrotic inflammation are quite variable in the lesional skin of individual patients with SSc owing to the diverse heterogeneity of this disease, even though patients with similar clinical backgrounds are selected. According to a couple of previous reports, the expression of Th17 cytokines is elevated in the lesional skin of patients with early SSc and positively correlates with the severity of skin sclerosis [25, 48–50]. The reproducibility of these results among several independent studies strongly indicates a critical role of Th17 cytokines in skin fibrosis of SSc. Taking into account the present data indicating that Fli1 haploinsufficiency increases the proportion of Th17-like Tregs under profibrotic inflammatory conditions, Th17-like Tregs are potentially involved in the development of skin fibrosis to variable degrees in individual SSc cases.

## Conclusions

To our knowledge, this is the first report demonstrating that Fli1 haploinsufficiency increases Th2- and Th17-like Treg proportions in BLM-induced profibrotic skin condition, in which IL-33-producing dermal fibroblasts contribute to Th2-like Treg transdifferentiation. The present findings indicate that IL-33-dependent transdifferentiation of Th2-like Tregs in SSc lesional skin, a notion proposed by MacDonald et al. [2], is part of an Fli1 deficiency-dependent pathological process. Overall, these data further strengthen the importance of Fli1 deficiency in the developmental process of SSc and reinforce the notion that dermal fibroblast-dependent regulation of immune cell phenotypes is involved in the development of skin fibrosis in SSc.

## Additional files

Additional file 1: Figure S1. Scatterplots of Fig. 1b. Scatterplots of the proportions of IL-4-, IL-13-, IL17A-, and IFN-γ-producing splenic Tregs from bleomycin (BLM)-treated wild-type and *Fli1*^*+/−*^ mice (*n* = 6). (PDF 592 kb)

Additional file 2: Figure S2. Signal intensity of IL-33. Signal intensity of IL-33 was analyzed and summarized. To quantify signal intensity of IL-33, color images were converted to grayscale, and then the brightness was measured in five different randomly selected fibroblasts and epidermal areas per specimen. *WT* Wild-type mice; *Basal* Under physiological condition; *BLM* Bleomycin-treated. (PDF 57 kb)

Additional file 3: Figure S3. Original films of immunoblotting with molecular weight markers. Original films of Figs. 2e and 4a. Molecular weight markers are shown. (PDF 573 kb)

## Abbreviations

AU: Arbitrary units
BLM: Bleomycin
ChIP: Chromatin immunoprecipitation
dcSSc: Diffuse cutaneous systemic sclerosis
Fli1: Friend leukemia virus integration 1
IFN: Interferon
IgG: Immunoglobulin G
IL: Interleukin
MFI: Mean fluorescence intensity
mRNA: Messenger RNA
Treg: Regulatory T cell
SSc: Systemic sclerosis
SSC-A: Side scatter area
TGF-β: Transforming growth factor-β
Th: T helper cell
TNF-α: Tumor necrosis factor-α
WT: Wild type
