# Supplementary figures and images for: Fli1-haploinsufficient dermal fibroblasts promote skin-localized transdifferentiation of Th2-like regulatory T cells
Source: Arthritis Res Ther. 2018 Feb 7;20:23. doi: 10.1186/s13075-018-1521-3 (PMC5803841; doi:10.1186/s13075-018-1521-3)

Supplementary Fig. 1

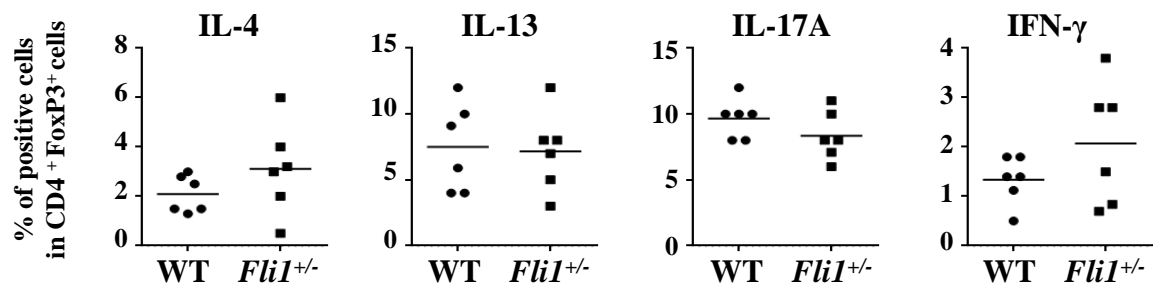

Supplement: Supplementary file 1 — Scatterplots of Fig. 1b. Scatterplots of the proportions of IL-4-, IL-13-, IL17A-, and IFN-γ-producing splenic Tregs from bleomycin (BLM)-treated wild-type and Fli1+/− mice (n = 6). (PDF 592 kb) [file 13075_2018_1521_MOESM1_ESM.pdf]

Supplementary Fig. 2

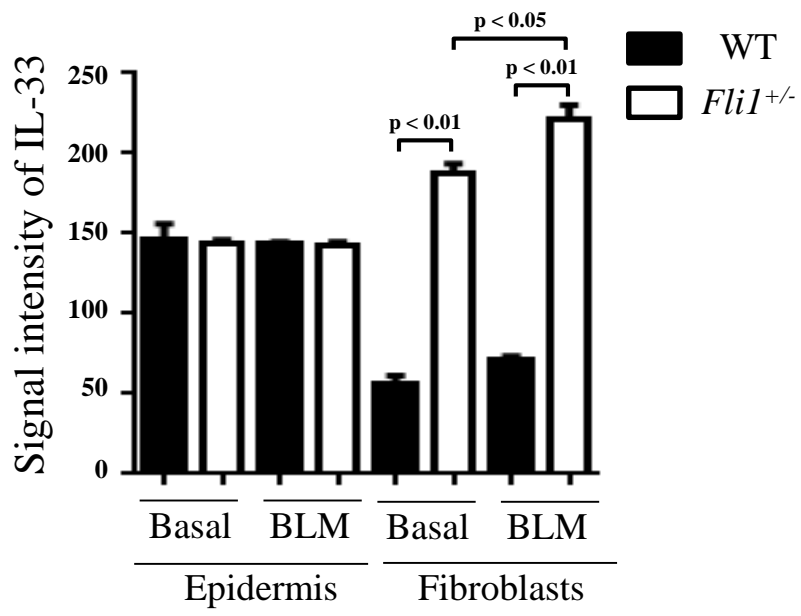

Supplement: Supplementary file 2 — Signal intensity of IL-33. Signal intensity of IL-33 was analyzed and summarized. To quantify signal intensity of IL-33, color images were converted to grayscale, and then the brightness was measured in five different randomly selected fibroblasts and epidermal areas per specimen. WT Wild-type mice; Basal Under physiological condition; BLM Bleomycin-treated. (PDF 57 kb) [file 13075_2018_1521_MOESM2_ESM.pdf]

Supplementary Fig. 3

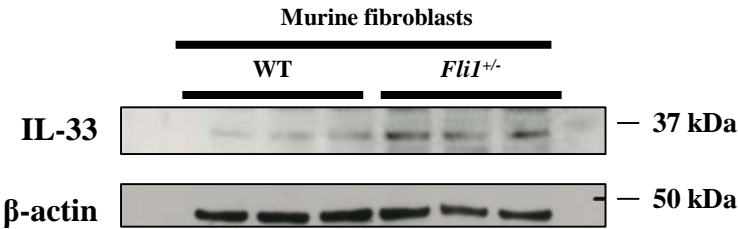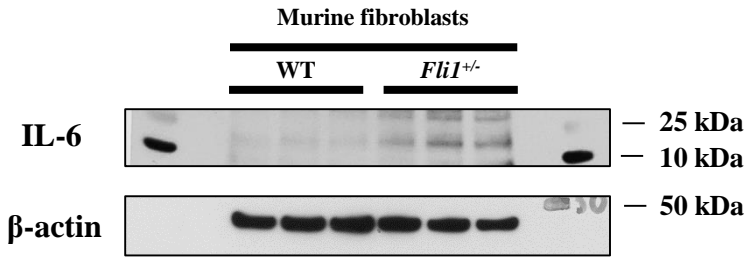

Supplement: Supplementary file 3 — Original films of immunoblotting with molecular weight markers. Original films of Figs. 2e and 4a. Molecular weight markers are shown. (PDF 573 kb) [file 13075_2018_1521_MOESM3_ESM.pdf]
